# Ultrasound-diagnosed disorders in shoulder patients in daily general practice: a retrospective observational study

**DOI:** 10.1186/1471-2296-15-115

**Published:** 2014-06-10

**Authors:** Ramon PG Ottenheijm, Inge GM van’t Klooster, Laurens MM Starmans, Kurt Vanderdood, Rob A de Bie, Geert-Jan Dinant, Jochen WL Cals

**Affiliations:** 1 Department of Family Medicine, CAPHRI School for Public Health and Primary Care, Maastricht University, PO Box 616, Maastricht, MD 6200, The Netherlands; 2 Department of Radiology, Orbis Medical Centre, Dr. H. van der Hoffplein 1, Sittard-Geleen, BG 6162, The Netherlands; 3 Department of Epidemiology, CAPHRI School for Public Health and Primary Care, Maastricht University, PO Box 616, Maastricht, MD 6200, The Netherlands

**Keywords:** Shoulder, Shoulder impingement syndrome, Rotator cuff, Ultrasound, General practice

## Abstract

**Background:**

Ultrasound imaging (US) is considered an accurate and widely available method to diagnose subacromial disorders. Yet, the frequency of the specific US-diagnosed shoulder disorders of patients with shoulder pain referred from general practice is unknown. We set out to determine the frequency of specific US-diagnosed shoulder disorders in daily practice in these patients and to investigate if the disorders detected differ between specific subgroups based on age and duration of pain.

**Methods:**

A predefined selection of 240 ultrasound reports of patients with shoulder pain (20 reports for each month in 2011) from a general hospital (Orbis Medical Centre Sittard-Geleen, The Netherlands) were descriptively analysed. Inclusion criteria were: (i) referral from general practice, (ii) age ≥18 years, and (iii) unilateral shoulder examination. Subgroups were created for age (<65 years and ≥65 years) and duration of pain (acute or subacute (<12 weeks) and chronic (≥12 weeks)). The occurrence of each specific disorder is expressed as absolute and relative frequencies.

**Results:**

With 29%, calcific tendonitis was the most frequently diagnosed disorder, followed by subacromial-subdeltoid bursitis (12%), tendinopathy (11%), partial-thickness tears (11%), full-thickness tears (8%) and AC-osteoarthritis (0.4%). For 40% of patients, no disorders were found on US. Significantly more full thickness-tears were found in the ≥65 years group. ‘No disorders’ was reported significantly more often in the <65 years group. The supraspinatus tendon was the most frequently affected tendon (72%).

**Conclusions:**

Calcific tendonitis is the most common US-diagnosed disorder affecting patients in general practice, followed by subacromial-subdeltoid bursitis, tendinopathy, partial- and full-thickness tears and AC-osteoarthritis. Full-thickness tears were diagnosed significantly more frequently in patients ≥65 years, while ‘no disorders’ was more frequently reported in patients <65 years. Our findings imply that patients can be stratified into diagnostic subgroups, allowing more tailored treatment than currently applied.

## Background

Shoulder pain is a common and disabling complaint in general practice with often a poor prognosis [1-4]. Almost 90% of patients with shoulder pain are diagnosed and treated in general practice, while only 10% are referred for a specialist opinion [5]. Accurately diagnosing patients with shoulder pain is, however, a complex problem. Tailoring treatment to the underlying disorder is difficult for general practitioners (GPs), as the medical history and physical examination do not provide conclusive evidence on the patho-anatomical origin of the symptoms [1]. Of the currently available additional diagnostic imaging tests, ultrasound imaging (US) is considered an accurate and widely available method to diagnose subacromial disorders [6-9].

Subacromial disorders are considered the most common pathology affecting the shoulder. The spectrum of subacromial pathology includes rotator cuff tendinopathy (tendinosis), calcific tendonitis, partial- and full-thickness tears, and subacromial-subdeltoid (SASD) bursitis [10-12]. Prevalence studies of US diagnoses in patients with shoulder pain in secondary care have shown prevalences ranging from 30-39% for tendinopathy, 13-15% for calcific tendonitis, 13-51% for partial-thickness tears, 24-70% for full-thickness tears, and 12-56% for SASD bursitis [6]. Remarkably, the prevalence of these disorders in general practice is still unknown. Unravelling the current case mix of patients with shoulder pain seen in general practice should give GPs more insight into the underlying causes of shoulder pain. The first step in providing this insight is to evaluate current daily practice.

The primary objective of this study was to determine the frequency of the specific US-diagnosed shoulder disorders of patients with shoulder pain referred from general practice. Additionally, we investigated which tendons (rotator cuff and longhead of the biceps) were affected, and if the frequencies differed between specific subgroups based on age and duration of pain.

## Methods

We conducted a retrospective observational study on a selection of reports of US shoulder examinations performed at the Orbis Medical Centre (OMC), a general hospital in the south of the Netherlands, in 2011. The OMC’s catchment area includes approximately 80 GPs (approximately 200,000 people) who refer their patients for US of the shoulder to this hospital through direct access. The study was approved by the Atrium - Orbis - Zuyd Medical Ethics Committee. Informed consent from patients was not needed as this was a retrospective chart study and data were analysed anonymously.

The OMC radiology department annually performs approximately 2400 US examinations of the shoulder for general practice patients. We expected a frequency of 24% for calcific tendonitis, based on the only available study with patients recruited from primary care [13]. Based on a 95% confidence interval and a precision of 5%, this resulted in a required sample size of 252 [14]. We therefore selected a predefined sample of 10% of the total of 2400 reports from the OMC database, stratified by month (January – December) to ensure equal distribution across seasons, resulting in a total sample of 240 reports. As degeneration is one of the mechanisms leading to rotator cuff pathology, and therefore a potential confounder, we decided also to stratify for age. Since approximately 10% of all shoulder US examinations are performed on patients ≥65 years, we decided that 10% of all reports per month had to be of patients ≥65 years. Hence, we analyzed the first 18 reports of each month of patients <65 years and the first two reports of patients ≥65 years. We realise that degeneration is a continuum, and evidence for a clear cut-off value for age as a confounder is lacking. Although arbitrary, we think it is plausible to set this cut-off for age at 65 years. General inclusion criteria were: (i) patients with shoulder pain referred by their GP (ii) age ≥18 years, and (iii) unilateral shoulder examination.

All US examinations were performed by an ultrasonographer with >15 years of experience of shoulder US, using an international scanning protocol [15], and standardised US diagnostic criteria for pathology were used [16]. The long head of the biceps tendon (LHBT), rotator cuff, SASD bursa, deltoid muscle and acromioclavicular (AC)-joint were routinely evaluated. US examinations were performed using equipment manufactured by two vendors; Hitachi/Aloka and Philips. All units were equipped with 5–12 MHz broadband linear-array transducers.

Two researchers (IGMK and LMMS) independently assessed the US reports, including age, gender and affected shoulder, and each verified the data entered by the other. The first 32 cases were assessed with a third independent researcher (RPGO) to discuss and enhance consistent assessments. Disagreements between the two main assessors were discussed and solved by consensus. In cases of persistent disagreement (14 of 240 cases), the third rater resolved the disagreement. Relevant data were missing from five US reports. These were completed by contacting the ultrasonographer.

For the subsequent analyses, subgroups were created for age (<65 years and ≥65 years) and duration of complaints (acute or subacute (<12 weeks) and chronic (≥12 weeks)), based on the duration indicated by the GP in the letter of referral. We used 12 weeks as the cut-off value, as the guidelines for shoulder complaints of the Dutch College of General Practitioners recommend US of the shoulder only in case of complaints persisting despite conservative treatment [1]. Normally, this refers to a period of approximately 12 weeks. All phrases like ‘a couple’ and ‘some’ were consistently interpreted and transformed into duration of pain in weeks, e.g. ‘a couple of weeks’ into two weeks, and ‘chronic’ into ≥12 weeks.

We calculated proportions with corresponding 95% confidence intervals. The chi-squared test was used for subgroup analysis. Data were analysed using SPSS (version 19.0).

## Results

### Patient characteristics

Of the 240 included patients, 131 (55%) were women. Mean patient age was 51.5 years (SD 12.1, range 20–83 years). US was performed on the left shoulder in 125 (52%) patients and on the right shoulder in 115 (48%). In six US reports the ultrasonographer commented that US was less reliable due to adiposity (3) or restriction of movement (3). Duration of pain was mentioned in 33% of the reports (79/240), with a minimal duration of 1 week and a maximum of >3 years. The duration referred to acute or subacute complaints in 42% (33/79) of these reports, and to chronic complaints in 58% (46/79). Median duration was 12 weeks, with lower and upper quartiles of 6 and 14 weeks.

### Frequency of US-diagnosed shoulder disorders

In 40% of the patients (97/240), no disorders were found on US. Of the remaining 60% of the patients (143/240), multiple disorders were found in 40% (59/143). Of these, 41 patients had two disorders, 15 had three, two had four and one patient had five disorders described in the US report.

Table 1 shows the frequency of the diagnosed disorders. With 29%, calcific tendonitis was the most frequently diagnosed disorder (69/240), followed by SASD bursitis (12%, 29/240), tendinopathy (11%, 27/240), partial-thickness tears (11%, 27/240), full-thickness tears (8%, 20/240) and AC-osteoarthritis (0.4%, 1/240). Of the remaining patients with a disorder, 8% (18/240) had a diagnosis classified as ‘Other’ (enthesophyte (5), tenosynovitis of the LHBT (4), fracture of the greater tuberosity (2), AC-joint widening (2), post-op supraspinatus tendon repair (2), subluxation of the LHBT (1) and calcific deposit in deltoid muscle(1)).

**Table 1:** Frequency of specific US-diagnosed shoulder disorders including comparisons for the ‘age’ and ‘duration of pain’ subgroups

	**Total**		**Age**						**Duration of pain in weeks**					
	**n = 240**		**<65 (n = 216)**			**≥65 (n = 24)**			**<12 weeks (n = 33)**			**≥12 weeks (n = 46)**		
	**n**	**%**	**n**	**%**	**95% CI**	**n**	**%**	**95% CI**	**n**	**%**	**95% CI**	**n**	**%**	**95% CI**
Calcific tendonitis	69	28.8	65	30.1	23.9-36.7	4	16.6	0.6-32.7	5	15.2	2.2-28.1	12	26.1	12.9-39.3
SASD bursitis	29	12.1	26	12	7.7-16.4	3	12.5	−1.8-26.8	4	12.1	0.4-23.9	6	13	2.9-23.1
Tendinopathy	27	11.3	22	10.2	6.1-14.3	5	20.8	3.3-38.4	3	9.1	−1.3-19.4	3	6.5	0.9-13.9
Partial-thickness tear	27	11.3	23	10.7	6.5-14.8	4	16.6	0.6-32.7	5	15.2	2.2-28.1	6	13	2.9-23.2
Full-thickness tear	20	8.3	13	6	2.8-9.2*	7	29.2	9.6-48.8*	2	6.1	−2.5-14.7	2	4.3	−1.8-10.5
AC-osteoarthritis	1	0.4	1	0.5	−0.5-1.0	0	0		0	0		0	0	
Other	18	7.5	14	6.5	3.2-9.8	4	16.6	0.6-32.7	2	6.1	−2.5-14.7	2	4.3	−1.8-10.5
No disorder	97	40.4	92	42.6	40.0-49.2*	5	20.8	3.3-38.6*	16	48.5	30.5-66.5	22	47.8	32.8-62.8

SASD subacromial-subdeltoid; *AC* acromio-clavicular joint.

*Significant finding (p < 0.05).

Within the group of 41 patients with two disorders, calcific tendonitis was the most prevalent disorder (56%, 23/41), followed by tendinopathy (15%, 6/41). Calcific tendonitis was most often seen in combination with tendinopathy (34%, 14/41), followed by SASD bursitis (15%, 6/41).

Table 1 shows that significantly more full-thickness tears were found in the ≥65 years group, while ‘No disorders’ was reported significantly more often in the <65 years group. The frequency of the other specific disorders did not differ significantly between the subgroups.

### Tendons affected

As multiple disorders per patient could be recorded, the total number of affected tendons was 183 (Table 2). The supraspinatus tendon was the most frequently affected tendon (72% 132/183), followed by the subscapularis tendon (12%), infraspinatus tendon (12%) and the LHBT (4%). Forty-three percent (10/29) of patients with SASD bursitis had concomitant calcific tendonitis, with a specific calcific bursitis in 10% (3/29).

**Table 2:** Tendon location of different disorders

	**Calcific tendonitis**		**Tendinopathy**		**Partial-thickness tear**		**Full-thickness tear**		**Other disorder**		**Total tendons**	
	**n = 94**		**n = 28**		**n = 28**		**n = 22**		**n = 11**		**n = 183**	
	**n**	**%**	**n**	**%**	**n**	**%**	**n**	**%**	**n**	**%**	**n**	**%**
SSP	58	61.7	26	92.9	26	92.9	17	77.3	5	45.5	132	72.1
ISP	19	20.2	0	0	1	3.6	1	4.5	1	0.9	22	12.0
SSC	17	18.1	1	3.6	1	3.6	3	13.6	0	0	22	12.0
LHBT	0	0	1	3.6	0	0	1	4.5	5	45.5	7	3.8

*SSP* supraspinatus tendon; *ISP* infraspinatus tendon; *SSC* subscapularis tendon; *LHBT* long head biceps tendon.

## Discussion

### Main findings

This study is the first to describe specific shoulder disorders detected on US in general practice patients who are symptomatic enough and have had symptoms for a sufficient duration of time to warrant referral by their GP for US. Calcific tendonitis, also referred to as calcific tendinopathy, was the most common US-diagnosed disorder affecting these general practice patients, followed by SASD bursitis, tendinopathy, partial-and full-thickness tears and AC-osteoarthritis. We found that full-thickness tears were diagnosed more frequently in patients ≥65 years, while patients <65 years were more often reported to have ‘no disorders’. Prevalence of specific shoulder disorders did not differ between patients with acute or subacute complaints or chronic pain. The supraspinatus tendon was the most commonly affected tendon.

### Strengths and limitations of this study

This study was the first to describe specific shoulder disorders detected on US in general practice patients. As almost 90% of the patients with shoulder pain are diagnosed and treated in general practice [5], these results are useful in the debate about the role of US in the diagnostic work-up of patients and about tailoring treatment to the underlying disorder, as well as in designing new studies.

We carried out a retrospective observational study on a selection of 10% (n = 240) of the US reports made in one year. The retrospective nature as well as the size of our sample and sampling method may have caused bias. As daily practice was the scope of our study, we only studied those patients for whom the GP ordered US. We realise that our secondary subgroup analyses were probably underpowered, especially for the ‘duration of pain’ subgroups, as duration was mentioned in only 33% of the reports. This may also have caused some selection and measurement bias limiting the interpretations. However, we can only speculate about the reasons for not reporting duration. Although the reliability of US examinations by a single ultrasonographer can be questioned, this situation is representative of daily practice. As the interrater-reliability between ultrasonographers examining patients with shoulder pain is high [17,18], a predefined scanning protocol was used**,** and the ultrasonographer was unaware of this planned study, the additional value of a second independent ultrasonographer would have been doubtful. Dynamic examinations to reveal impingement or adhesive capsulitis were not performed by the ultrasonographer. This might have resulted in an underrepresentation of adhesive capsulitis given the three patients with a restricted range of motion. To enhance consistent assessment, two researchers independently assessed the US reports and each verified the data entered by the other researcher. To correct for seasonal influences, e.g. traumatic rotator cuff tears in winter, we selected 20 reports from each month.

### Comparison with existing literature

Our results are not in line with previous secondary care studies, which showed lower prevalence of calcific tendonitis and higher prevalence of bursitis and the degenerative disorders tendinopathy and tendon tears [6,7]. This difference may be explained by the difference in patient setting and the resulting case mix. Previous studies were conducted in patients with mostly chronic shoulder pain consulting in secondary care settings, mostly in the diagnostic work-up for surgery. Referral to secondary care in the Netherlands is done by GPs, and patients are typically only referred for US in case of persistent complaints despite conservative treatment. Another explanation could be an overrepresentation in our study of patients with acute flairs of calcific tendonitis. This results in a different case mix compared to secondary care, where tendon tears are most prevalent.

The only study conducted in a primary care setting also found calcific tendonitis to be the most prevalent disorder (24%) [13]. However, that study found more partial-thickness tears (23%) and fewer patients with no disorder detected on US (15%). This can be explained by the difference in study design, patient setting and patient characteristics; the study was part of a wider prospective diagnostic accuracy study, with 98% of the patients recruited from physiotherapy practice and 93% of the recruited patients involved in a liability procedure because of an accident.

Our results show that the supraspinatus tendon was the most commonly affected tendon. This is in agreement with previous findings [13] and the general belief that the supraspinatus tendon is the tendon most commonly involved in rotator cuff disease [19].

### Implications for practice and research

As US is an easily accessible, accurate, relatively cheap and non-invasive and non-ionising imaging procedure, GPs in the study area frequently refer patients for US, resulting in a rise in test volume of 100% over the last 6 years. However, at present there is no clear evidence for using US for acute shoulder pain. The present study shows that different subacromial disorders can be detected in patients with shoulder pain in general practice. This implies that patients can be stratified into diagnostic subgroups, allowing more tailored treatment than currently applied. In general practice patients with low back pain, a stratified management approach combining prognostic screening and treatment targeting was found to improve patient outcome [20]. In daily practice, combining clinical information with US findings could potentially be helpful to tailor the treatment of patients with shoulder pain. It is likely that patients will respond best to interventions that address the etiology, affected structures, impairments, and relevant biomechanics that are specific to their diagnosis. However, there is no current general practice evidence to back this up [16].

Since a certain percentage of the US findings found is not responsible for the reported complaints [21,22], GPs should realise that there might not be a straightforward correlation between US and clinical findings. A prospective study of US findings with clinical input is necessary to provide a more robust indication of the true prevalence in general practice.

Calcific tendonitis was found to be the most prevalent disorder in our study, affecting about one-third of all patients. Calcific tendonitis of the shoulder is a common painful disorder characterised by calcifications in rotator cuff tendons, resulting in repeating painful episodes. The pathogenesis is still a matter of controversy. The most dominant view states that the deposition of calcium crystals within the substance of the tendon is not a degenerative process but one that is actively mediated by cells in a viable tendon. A natural cycle of repair exists, but this cycle can be blocked or delayed [23-25]. About 50% of patients with rotator cuff calcifications have shoulder pain, meaning that many individuals with radiographic evidence of calcific tendonitis are asymptomatic. It can be helpful if US reports contain information about size and location, as well as morphologic shape of the calcifications, as this might predict symptomatology. For instance, larger calcifications, those located at the confluence of supraspinatus and infraspinatus tendons, and calcifications in the resorptive phase tend to be more symptomatic [23,25]. Given this natural recovery cycle and asymptomatic patients, the question arises if all patients in our study with calcific tendonitis actually suffered from these calcification deposits and needed treatment. It is unknown if the same goes for the other disorders, especially in patients with more than one disorder.

By contrast, 40% of the patients in our study had no disorders detected on US. It is not surprising that the majority of these patients (95%) were <65 years. Hence, GPs may use US to diagnose disorders, but also to rule out specific shoulder disorders. In comparison to the elderly, younger patients are more physically active and may therefore experience more disability when faced with pain. It is our experience that younger patients with chronic shoulder pain in particular often ask for imaging to ensure that no diagnosis is missed. One should keep in mind that shoulder pain can also be caused by referred pain. Also, it is to be expected that tears of the rotator cuff occur more frequently in elderly patients, as a result of age-related degenerative tendon changes.

## Conclusions

This study shows that, contrary to findings from secondary care, calcific tendonitis is the most common US diagnosed disorder affecting patients who are symptomatic enough and have had symptoms for a sufficient duration to warrant referral by their GP for US. Full-thickness tears were diagnosed more frequently in patients ≥65 years. Our findings imply that patients can be stratified into diagnostic subgroups, allowing more tailored treatment than currently applied.

## Competing interests

The authors declare that they have no competing interests.

## Authors’ contributions

RPGO, IGMK, LMMS, RAB, GJD and JWLC participated in the study concept and design. RPGO, IGMK and LMMS participated in the acquisition of data and the statistical analysis. RPGO, IGMK, LMMS, KV, RAB, GJD and JWLC contributed to the analysis and interpretation of data. RPGO, IGMK, LMMS and JWLC participated in the drafting of the manuscript. RPGO, IGMK, LMMS, KV, RAB, GJD and JWLC participated in the critical revision of the manuscript for important intellectual content. All authors read and approved the final manuscript.

## Pre-publication history

The pre-publication history for this paper can be accessed here:

http://www.biomedcentral.com/1471-2296/15/115/prepub
